# Physiological and Genomic Analysis of *Bacillus pumilus* UAMX Isolated from the Gastrointestinal Tract of Overweight Individuals

**DOI:** 10.3390/microorganisms9051076

**Published:** 2021-05-17

**Authors:** José Luis Reyes-Cortes, Alejandro Azaola-Espinosa, Luis Lozano-Aguirre, Edith Ponce-Alquicira

**Affiliations:** 1 Departamento de Biotecnología, Universidad Autónoma Metropolitana Unidad Iztapalapa, Av. San Rafael Atlixco 186, Col. Vicentina, Ciudad de México 09340, Mexico; jose.luis.37@hotmail.com; 2 Departamento de Sistemas Biológicos, Universidad Autónoma Metropolitana Unidad Xochimilco, Calzada del Hueso 1100, Coyoacán, Ciudad de México 04960, Mexico; azaola@correo.xoc.uam.mx; 3 Unidad de Análisis Bioinformáticos del Centro de Ciencias Genómicas, UNAM, Cuernavaca, Morelos 62210, Mexico; llozano@ccg.unam.mx

**Keywords:** microbiota, pangenome, phylogenomic and bacillus

## Abstract

The study aimed to evaluate the metabolism and resistance to the gastrointestinal tract conditions of *Bacillus pumilus* UAMX (BP-UAMX) isolated from overweight individuals using genomic tools. Specifically, we assessed its ability to metabolize various carbon sources, its resistance to low pH exposure, and its growth in the presence of bile salts. The genomic and bioinformatic analyses included the prediction of gene and protein metabolic functions, a pan-genome and phylogenomic analysis. BP-UAMX survived at pH 3, while bile salts (0.2–0.3% *w/v*) increased its growth rate. Moreover, it showed the ability to metabolize simple and complex carbon sources (glucose, starch, carboxymethyl-cellulose, inulin, and tributyrin), showing a differentiated electrophoretic profile. Genome was assembled into a single contig, with a high percentage of genes and proteins associated with the metabolism of amino acids, carbohydrates, and lipids. Antibiotic resistance genes were detected, but only one beta-Lactam resistance protein related to the inhibition of peptidoglycan biosynthesis was identified. The pan-genome of BP-UAMX is still open with phylogenetic similarities with other *Bacillus* of human origin. Therefore, BP-UAMX seems to be adapted to the intestinal environment, with physiological and genomic analyses demonstrating the ability to metabolize complex carbon sources, the strain has an open pan-genome with continuous evolution and adaptation.

## 1. Introduction

Gram-positive *Bacillus pumilus* are spore-forming bacteria that grow under aerobic or anaerobic conditions. These bacteria have been reported to be found in soil, water, air (spores), fermented foods, decomposing plant and animal tissues, and the human gastrointestinal tract (GIT). The *Bacillus* genus is not considered as part of the normal microbiota composition in the human gut. However, recent studies have shown that *Bacillus* may be present in the GIT in high amounts when associated with food intake [1,2,3,4], which is related to the identification of *B. pumilus*, *B. licheniformis*, *B. clausii*, *B. subtilis*, *B. megaterium*, *B. mediterraneensis*, and *B. thuringiensis* from the human GIT [1,5,6]. However, the environmental and nutritional conditions that vary throughout the GIT and the presence of other microorganisms can restrict *Bacillus* colonization. Their survival depends on the availability of specific carbon sources (provided by the diet of the individual) and on the activity of the various enzymes that are present in saliva and throughout the GIT. In addition, pH variation also affects the availability of the carbon source. For example, the stomach is an acidic environment (pH 1–3) owing to the presence of gastric acid. However, the small intestine (pH close to 7–8) and the colon (pH 7–9) present more alkaline conditions [7,8]. Bacteria from the *Bacillus* genus (such as *B. pumilus*) can produce spores that may be responsible for its survival in the acidic gastric barrier [2,9,10,11]. The resistance of some Bacillus strain to the GIT conditions has led to their consideration and application as probiotics [12,13]. Several *Bacillus* strains have a long history of safe use in fermented foods, and well known for their ability to secrete proteins, enzymes, antimicrobial compounds, and vitamins, which make this group a candidate to be used as probiotic bacteria in the food and pharmaceutical industries [14]. In particular, the Bacillus group produces food grade degrading enzymes, and have been employed for the production of nutraceuticals, including riboflavin, cobalamin, carotenoids as supplements for human consumption [15]. Moreover, it has been reported that the *Bacillus* species had a broader activity against various cancer cells in the mammary gland, colon, and liver [16,17], and are capable of producing inhibitory compounds against *Helicobacter pylori*, fungi, and Gram-positive bacteria [14,18]. While *B. pumilus* among other Bacillus can produce enzymes that hydrolyze gluten to non-immunogenic peptides that protect the intestinal mucosa and gamma-aminobutyric acid [16,19]. Recently, the protease activity of this genera has been related to the production of protein compounds that alleviate the progression and aggravation of Parkinson Disease, and have more beneficial effects for human health, including antioxidant activity and the regulation of the redox balance [20]. Furthermore, *Bacillus* have the capacity of producing short-chain fatty acids (SCFA), such as acetic and propionic acid, and mainly butyric acid which can reduce appetite and significantly reduce body weight in obese individuals [21]. However, an unbalance in the GUT microbial population has been related to the development of obesity, diabetes, and liver diseases, derived from the monosaccharides and FA within the GIT that provides additionally energy to the host, and the increased intestinal permeability due to the production lipopolysaccharides in response to the consumption of high-fat diets [22,23]. Nonetheless, few members of the *Bacillus* spp. group such as *B. cereus* and *B. thuringensis* are related to food borne intoxications. Therefore, the use of genotypic and phenotypic tools has a great relevance for the selection of individual *Bacillus* strains and to evaluate their beneficial functionality as potential probiotics [15].

Fakhry and Leser et al. proposed that the resistance of Bacillus spores and vegetative cells to simulated gastric conditions could result from differential gene expression as a means of adaption to the GIT environment [1,14].

Omic technologies such as PacBio genome sequencing allow the analysis of the gene expression variation which can provide information in a shorter time frame [24,25,26,27,28,29,30]. Genomic sequencing provides a complete catalog of the expressed genes in the microorganism and the identification of its virulence factors. Furthermore, genome sequencing allows for a comparative phylogenetic analysis, where encoded proteins are rationally classified according to the Clusters of Orthologous Groups (COGs) database, in order to assess their functionality and evolution [28,29,30,31,32]. In addition, the study of metabolic networks through online databases, such as the KEGG Automatic Annotation Server (KAAS) [33], allows the reconstruction of metabolic pathways based on sequence homology, bidirectional information, and heuristics with a high degree of accuracy [34,35]. Finally, these tools also offer other elements that contribute to the study of gene functionality in bacteria, such as pan-genome analysis. In some species, it has been shown that new genes could be discovered even after the genome was fully sequenced. Therefore, the pan-genome analysis can estimate the number of additional complete genome sequences that are required to characterize the entire genomic diversity [36,37,38].

## 2. Materials and Methods

### 2.1. Strain Propagation

BP-UAMX was previously isolated by Mayorga [4] from fecal samples of overweight individuals (body max index: 27.17 ± 0.51 kg/m^2^). Prior to the analysis, the isolates were propagated in culture media (2.2 g/L NaCl, 10 g/L (NH_4_)_2_SO_4_, 0.4 g/L CaCl_2_, 0.4 g/L MgSO_4_, 0.72 g/L K_2_HPO_4_, 0.2 g/L yeast extract, and 10 g/L glucose) under continuous agitation (180 rpm), at 37 °C. All reagents were acquired from Sigma Chemical Co. (St. Louis, MO, USA).

### 2.2. GenBank Accession Number for the Studied Strain

The entire genome of the studied strain was sequenced and deposited in GenBank (NCBI) with the assigned accession number: “*Bacillus pumilus* str. UAMX isolate”: CP058951 (https://www.ncbi.nlm.nih.gov/nuccore/CP058951.1/).

### 2.3. Resistance Assessment to Simulated GIT Physiological Conditions

The resistance of BP-UAMX to GIT physiological conditions was determined following the procedure described by Hernández-Alcántara [39]. Briefly, growth kinetics assays were performed after exposing the cultured bacteria to different pHs (2, 3, and 7 as ontrol) and bile salts concentrations (0.2 and 0.3% *w/v*), for 12 min and 3 h.

### 2.4. Growth Ability in Different Carbon Sources

To evaluate the effect of the carbon source on the metabolism of BP-UAMX, growth kinetics assays were performed in the presence of 0.2% starch (J.T. Baker, Phillipsburg, NJ, USA), inulin (Campos Azules, Guadalajara, Jalisco, Mexico), carboxymethyl-cellulose or CMC (J. T. Baker, Phillipsburg, NJ, USA), and tributyrin (Sigma-Aldrich, St. Louis, MO, USA). The control assay was performed with 1% glucose (BD Bioxon, Cuautitlán Izcalli, Mex, Mexico). Bacteria were cultivated in microtiter plates (37 °C for 40 h) and the optical density at 600 nm was monitored using a Synergy HT microplate reader (BioTek Instruments Inc., Winooski, VT, USA). The growth kinetics data were adjusted to the Verhulst-Pearl logistic model [40] (vegetative cell growth) and an inverted exponential growth model (death phase). Following this, kinetic parameters were calculated and statistically validated with NCSS 2007, using the one-way variance analysis (*p* ≤ 0.05) [41].

### 2.5. Electrophoretic Analysis of BP-UAMX Cultured with Different Carbon Sources

The SDS-PAGE analysis was performed to compare the soluble protein content of BP-UAMX cultured using different carbon sources. Culture samples were collected at the late logarithmic phase and centrifuged at 11,000 rpm (4 °C) for 10 min (Eppendorf 5810R, Hamburg, Germany), with consecutive washes using cold phosphate-buffered saline (PBS, pH 7.2). Following this, the cell pellets were re-suspended in 291 μL PBS (pH 7.2) and 9 μL 10% SDS. Cell rupture was performed using a 750 W Ultrasonic Homogenizer (Cole-Parmer, Vernon Hills, IL, USA) with 25 cycles of 45 s, followed by another centrifugation step (11,000 rpm for 20 min, at 4 °C). The soluble cytosolic protein fraction was recovered and quantified using the Bradford method [42]. Finally, SDS-PAGE was performed using 12% Tris-glycine-polyacrylamide gels and a MiniProtean 3 electrophoresis chamber (BioRad, Hercules, CA, USA), as described by Laemmli [43].

### 2.6. Proteomic Profiling Analysis for BP-UAMX

The proteomic analysis for the control culture of BP-UAMX growth in 1% glucose was performed as reported by Pérez-Acosta et al. [44]. Briefly, electrophorectic gels were excised and subjected to reduction (20 µL DTT 10 mM, NH_4_HCO_3_ 50 mM, for 45 min at 56 °C), alkylation (20 µL yodoacetamide 100 mM, NH_4_HCO_3_ 50 mM, 30 min), and digestion with trypsin (12.5 ng/μL Trypsin Gold; Promega, Madison, WI, USA, 16 h, 37 °C). After digestion and desalting, the peptides were analyzed in a nano-LC-MS/MS in a Orbitrap Fusoin^TM^ Tribid^TM^ mass spectrometer (Thermo-Fisher Scientific, San Jose, CA, USA) coupled with a UltiMate 3000 RSLC system (Dionex, Sunnyvale, CA, USA) and set with an EASY-Spray nano ion source (Thermo-Fisher San Jose, CA, USA). Each reconstitued sample (5 μL, formic acid 1%) was loaded into a nanoviper C18 trap column (3 μm, 75 μm × 2 cm, Dionex) at a 3 μL/min flow rate and separated on an EASY spray C18 RSLC column (2 µm, 75 µm × 25 cm), using a 100 min gradient with a flow rate of 300 nL/min, using set Solvent A (0.1% formic acid in LC-MS grade water) and Solvent B (0.1% formic acid in 90% acetonitrile). The gradient was as as follows: Solvent A for 10 min, 5–20% of Solvent B for 20 min, 25–95% of Solvent B for 5 min, 95% of Solvent B for 10 min, 95–5% of Solvent B for 5 min, and 8 min of Solvent A.

The mass spectrometer was operated in the positive ion mode with a nanospray voltage set at 3.5 kV and a source temperature at 280 °C. External calibrants included caffeine, Met-Arg-Phe-Ala (MRFA), and Ultramark 1621. Full MS scans were carried with 120,000 of resolution (FWHM), scan range of 350–1500 *m*/*z* and 10 ppm, and 0.2 Da mass tolerance. For MS2, the most abundant MS 1 were isolated with charge rates set between 2–5 with a mass range of 650–1200 *m*/*z*. Data acquisition was done using the software Xcalibur v4.0.27.10 (Thermo-Fisher Scientific). Raw data were procesed with Proteome Discovery v2.1 (PD, Thermo Fisher Scientific Inc.); then a subsequent search was carried with the Marcot search (v2.4.1, Matrix Science, Boston, MA, USA) within the revised data set Uniprot Viridiplantae (http://www.uniprot.org/proteomes/?query=viridiplantae+&sort=score, accessed on 12 March 2020) for *Bacillus pumilus*. Search parameteers included full-tryptic protease specificity and two missed cleavages. Fixed modifications included carbamidomethylation of cysteine and iTRAQ 4-plex N.terminal/lysine residues (+57.021 Da). Varibable modifications include methionine oxidaction (+15,995 Da) and deamidation in asparagine/glutamine (+0.984 Da). For the MS2 method, in which identification was performed at a high resolution in the Orbitrap, precursor ion tolerances and fragments of ±10 ppm and ±0.2 Da were applied.

Finnaly, the obtained proteins were also annotated using the COG-NCBI database for their grouping by function (http://www.ncbi.nlm.nih.gov/COG, accessed on 6 May 2021) [45]. Moreover, for the Kyoto Encyclopedia of Genes and Genomes (KASS-KEGG) to obtain the KEGG Ontology (the KO number sequence), the complete list was used for KEGG Maped to reconstruct the preducted pathways associated. (https://www.genome.jp/kegg/, accessed on 6 May 2021) [33,34,35,45,46].

### 2.7. Sequencing and Total Genome Assembly

Total DNA extraction was performed using a commercial kit (Promega, Woods Hollow Road, Madison, WI, USA), following the manufacturer’s specifications. The presence and integrity of DNA was confirmed using 0.8% agarose gel electrophoresis in a 10 mM tris-acetate buffer (pH 8) with 1 mM EDTA. DNA samples were also quantified, ensuring a concentration greater than 80 ng. The BP-UAMX genome was sequenced by Macrogen Inc., Seoul, Korea, using PacBio RS II technology, which generated libraries of 20 Kb [38].

De novo genome assembly was performed using three bioinformatic tools: Canu 1.8 [47], SMRTools 5.0.1, and Unicycler GPLv3 [48]. The quality of the assembly was evaluated using QUAST 5.0 [49]. Gene annotation was performed with Prokka GPLv3 [50]. Finally, the assembled genome was analyzed using the NCBI database.

### 2.8. Gene Grouping by Function and Metabolic Pathway Prediction

Gene groups were classified using the NCBI COGs database (http://www.ncbi.nlm.nih.gov/COG, accessed on 12 April 2021) [45], which was created for the phylogenetic classification of proteins obtained from complete genomes [45]. The metabolic pathway prediction was performed using the KEGG database (https://www.genome.jp/kegg/, accessed on 12 April 2021) [33,34,35,45,46].

### 2.9. Pan-Genome Analysis

The pan-genome was obtained using the software GET_HOMOLOGUES [51] and GET_PHYLOMARKERS [52]. First, 38 complete genomes of *B. pumilus* were randomly selected from the NCBI database. Following this, a pan-genome tree was built using 30 genomes of *B. subtilis* (phylogenetically related to *B. pumilus*) and 25 genomes of *Lysinibacillus* (an unrelated genus).

### 2.10. Statistical Analysis

All the experiments were performed in triplicate, results were expressed as the mean ± standard deviation. The significant differences were calculated using the Student’s *t* test, and the one-way analysis of variance (ANOVA) using the Tukey’s-test (*p* ≤ 0.05). All the calculations were determined using the NCSS 2007^®^ software [53].

## 3. Results and Discussion

### 3.1. Resistance to Gastrointestinal Conditions

**Figure 1:**
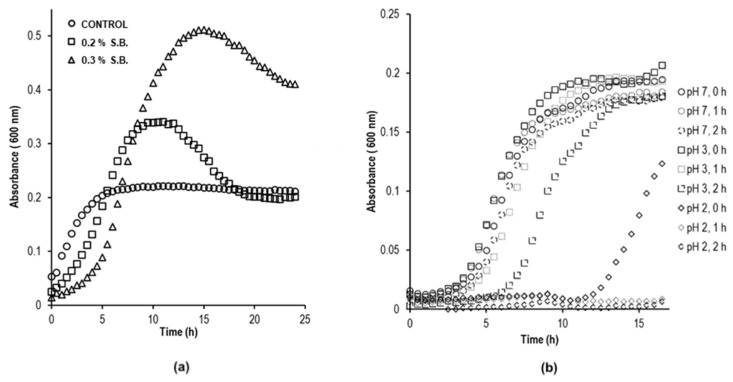
Kinetic growth for *B. pumilus* UAMX in the presence of (**a**) bile salts 0, 0.2 or 0.3% and (**b**) after exposure to pH (2, 3 and 7) for up to 2 h. Data were adjusted as indicated by the experimental procedures.

A combination of in vitro and in vivo studies is highly recommended for a better comprehension of GIT microbiota. However, in vitro studies are fully reproducible, rapid, less expensive, and do not involve ethical restrictions. Nonetheless, a variation in the experimental conditions may restrict data comparison within researchers [54,55,56]. Still, in vitro models are recommended for metabolic and biochemical experiments [57]. Therefore, this study was undertaken using an in vitro model based on the ability of *B. pumilus* UAMX (BP-UAMX) to survive at low pH or in the presence of bile salts that are related to the gastrointestinal transit from the gastric to the enteric sections, since the studied strain was previously isolated from the gut of overweight individuals. BP-UAMX isolates were cultivated after being exposed to bile salts (0.2 and 0.3% *w/v*) or to low pHs (2 and 3), according to the methodologies reported by Kristoffersen and Dressman et al., respectively [58,59]. The addition of bile salts induced an increase in microbial growth as shown in Figure 1a for the kinetic parameters OD_max_ (control: 0.222 ± 0.01; 0.2% *w/v* bile salts: 0.364 ± 0.02; 0.3% *w/v* bile salts: 0.517 ± 0.024) and microbial growth rate μ_max_ (control: 0.773 ± 0.0114 h^−1^; 0.2% *w/v* bile salts: 0.563 ± 0.018 h^−1^; 0.3% *w/v* bile salts: 0.567 ± 0.029 h^−1^). Therefore, not only did BP-UAMX resist exposure to bile salts, as reported by Berthold-Pluta et al. [60], but also, its growth was stimulated under such conditions. Alternatively, we observed variations in acid tolerance response as an adaptive mechanism for bacterial protection. Upon exposure to low pH, BP-UAMX microbial growth was significantly reduced. Exposure to pH 3 for 2 h led to a decrease in microbial growth (Figure 1b), as indicated by the lower kinetic descriptors OD_max_ (0.180 ± 0.009) and μ_max_ (0.433 ± 0.029 h^−1^), while 1 h of exposure to pH 2 induced total growth inhibition. Similar findings were reported by Berthold-Pluta for *B. cereus* [60]. However, Bacillus strains have shown higher survival response than other *Bifidobacterium* and *Lactobacillus* probiotic strains in simulated GIT conditions as reported by Soares et al. [61]. The data suggest the resistance of the studied strain to the GIT transit, but further studies need to be conducted as this simplified in vitro model has several restrictions in comparison to the in vivo conditions, where factors such as the GIT human physiology and peristaltic movements; the presence of gastric and pancreatic enzymes (proteases, amylases, and lipases), salts and hormones; as well as, the microbial capability for adhesion to the intestinal epithelium and their ability to proliferate in the presence of other microorganisms, are not considered [54,55,56].

### 3.2. Analysis of Growth Kinetics Using Different Carbon Sources

The growth kinetics of BP-UAMX was analyzed using different carbon sources: Glucose, starch, inulin, carboxymethyl cellulose (CMC), and tributyrin. The collected data were adjusted to the Verhulst-Pearl logistic model (Equation (1)) for vegetative cell growth, while an inverted exponential growth model (Equation (2)) was used to adjust the death phase data. The adjustment correlation coefficients were greater than 0.95.

(1) dx/dt=μx(1−x/x_m)

(2) dx/dt=−μx

**Figure 2:**
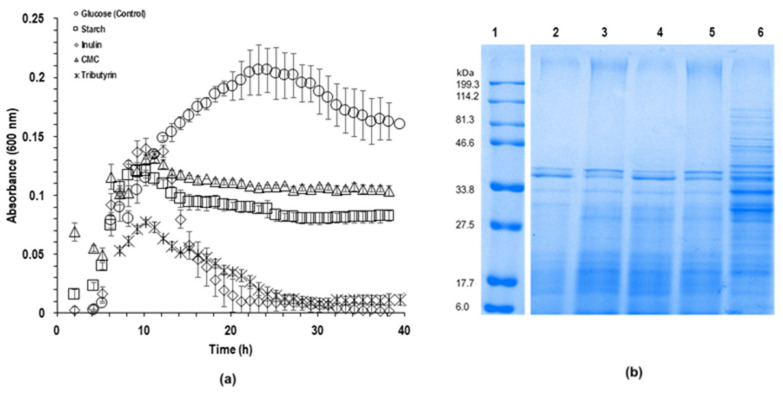
Influence of the carbon source on growth and protein expression. (**a**) Kinetic growth for *B. pumilus* UAMX under different carbon sources; (**b**) tris-glicine SDS-PAGE 12% for soluble cytosolic protein fraction of *B. pumilus* UAMX [line 1 (Broad Range MW standard, BioRad, Hercules, CA, USA), line 2 (glucose), line 3 (starch), line 4 (inuline), line 5 (CMC), and line 6 (tributyrin)].

BP-UAMX was able to metabolize all the carbon sources, as demonstrated by the growth curves in Figure 2a. The highest biomass was achieved in the presence of glucose (OD_max_ = 0.201 ± 0.019), which could be explained by this carbon source being easier to metabolize. Cultivation in the presence of complex carbon sources such as CMC (OD_max_ = 0.172 ± 0.01), starch (OD_max_ = 0.140 ± 0.01) or inulin (OD_max_ = 0.137 ± 0.006) resulted in lower biomass yields. In addition, the stationary phase lasted up to 40 h when CMC and starch were used as carbon sources. However, the addition of tributyrin led to a reduction in microbial growth (OD_max_ = 0.07 ± 0.001), even though lipolytic activity was reported in strains of *B. subtilis* [62,63,64]. Both inulin and tributyrin induced rapid cellular differentiation after 10 h of cultivation. Mallozzi and Handtke et al. reported similar findings and claimed that variations in the carbon source type and concentration might affect sporulation and biomass yield [65,66]. Cell lysis has also been associated with the production of esterases by *Bacillus* strains with lipolytic activity [59,60,61,62]. Therefore, while observing the death of vegetative cells after 10 h of cultivation in the presence of inulin and tributyrin, the sporulation process is likely to be initiated as a survival response [1,14,67]. Furthermore, Mallozzi and Handtke et al. stated that sporulation is an alternative mechanism for *B. pumilus* cells to cope with exposure to non-glycolytic carbon sources and stress, which could be attributed to an adaptation process [62,63,65]. Mukhopadhya et al. claimed that spore formation could be a critical factor that explained the occurrence of highly specialized bacteria in the gut microbiota, such as the starch metabolizing *Ruminicoccu brommi* [68]. Therefore, the growth of BP-UAMX in the presence of tributyrin may be associated with an adaptation process, since the strain was isolated from the microbiota of individuals that are prone to consuming a diet rich in sugar and fat [4]. In addition, Ferrer et al. reported that the phylum *Firmicutes* was more abundant than *Bacteroidetes* in the gut microbiota of obese adolescents when compared to lean individuals of the same age group [69].

### 3.3. Electrophoretic Analysis of BP-UAMX Cultured with Different Carbon Sources

For each carbon source, a BP-UAMX culture sample was collected at the late logarithmic phase, followed by total protein extraction. The SDS-PAGE profiles of cultures grown in the presence of glucose, starch, CMC, and inulin showed a similar pattern (Figure 2b, lines 2–5).

The electrophoretic profiles were mainly composed of protein bands with a molecular weight lower than 46.6 kDa, with a variation in intensity. However, the protein profile of the culture grown in the presence of tributyrin (Figure 2b, line 6) was quite different, showing protein bands up to 114.2 kDa. This could be explained by a differential protein expression derived from various processes such as membrane fluidity, lysis, and sporulation encouraged under stressing conditions, which were more evident in the presence of tributyrin, since the source of fatty acids is an important effector within the metabolism regulation and adaptation [70,71,72,73]. The increase in expressed proteins associated with fatty acid metabolism may provide access to alternative carbon sources when glucose or other comparable carbohydrates are not available [70,71]. Therefore, exposing BP-UAMX to non-glycolytic carbon sources results in new alternatives that should be addressed in the future.

A total of 334 proteins were identified and grouped by biological process functionality and cellular location, 195 proteins were related to the cytosol and 272 related to the membrane. Table 1 shows the ontology of proteins expressed by *Bacillus pumilus* UAMX growth on the glucose carbon source control media. The list of proteins and KEGG-PATHWAY database can be found in the Supplementary File. Most identified functional proteins were associated with the transport of carbohydrates, energy production, as well as to metabolic and structural intermediaries for the increase of cellular biomass, that could be associated with the observed growth and cell production rates in Figure 2a. It is well known that the *Bacillus* genus produces several enzymes of industrial interest, but lipases are produced in small amounts that coincide with the obtained proteomic approach of few proteins associated with the lipid metabolism [72]. Lipids play a role as a source of energy and membrane fluidity for the optimal growth, sporulation, and survival of *Bacillus*.

The fatty acid biosynthesis is an energy consumer and highly regulated process that allows maintaining an exact membrane composition that varies with the physiological cell state and with the lipid source as a response to the environmental conditions, including the growth media, temperature, oxygen, and pH [73]. The Bacillus membrane has a high content of branched (12–17 carbons), unsaturated, and complex fatty acids and their biosinthesys is vital. For instance, *Bacillus cereus* is capable of integrating exogenous free fatty acids into the cell membrane as an adaptation response to low temperatures and anaerobiosis. Conversely, some exogenous lipids such as monoglycerides, linolenic, palmitic, and stearic acids can also inhibit the growth of vegetative cells by decreasing the intracellular ATP level. Therefore, exogenous lipids can induce a positive or negative effect on vegetative cells and spore germination [73,74,75,76]. Thus, the reduction of BP-UAMX vegetative cells after 10 h of cultivation in the presence of inulin and tributyrin, may be related to the initiation of the sporulation or as an adaptation response. Furthermore, the microbiota bioactivity and bioavailability of nutrients are also correlated to the interaction within the GIT microbiota pattern to support their physiological functions, which was not considered under this experimental model [75].

### 3.4. Genome Sequencing and Assembly

The entire genome of BP-UAMX was sequenced (https://www.ncbi.nlm.nih.gov/nuccore/CP058951.1/ accessed 12 April 2021), obtaining a read quality of 0.85, an N50 value greater than 19,050 and a library size of 982 Mb. Following this, genome assembly was performed using Canu, SMRTools, and Unicycler, which are software tools that are commonly used in single molecule real-time sequencing technologies (SMRT). The best assembly was obtained with Canu, which allowed us to close the whole genome of BP-UAMX in a single contig of 3.85 Mb (N50: 3.85 Mb). A total of 3192 coding sequences (CDS) were identified (24 rRNA, 81 tRNA, and 1 tmRNA), with a GC content of 42%. In particular, the GC content may vary between species and strains due to factors such as genome size, mutations, and environmental conditions including temperature, aerobiosis, and nitrogen availability [76,77,78,79,80,81].

### 3.5. Pan-Genome Analysis

**Figure 3:**
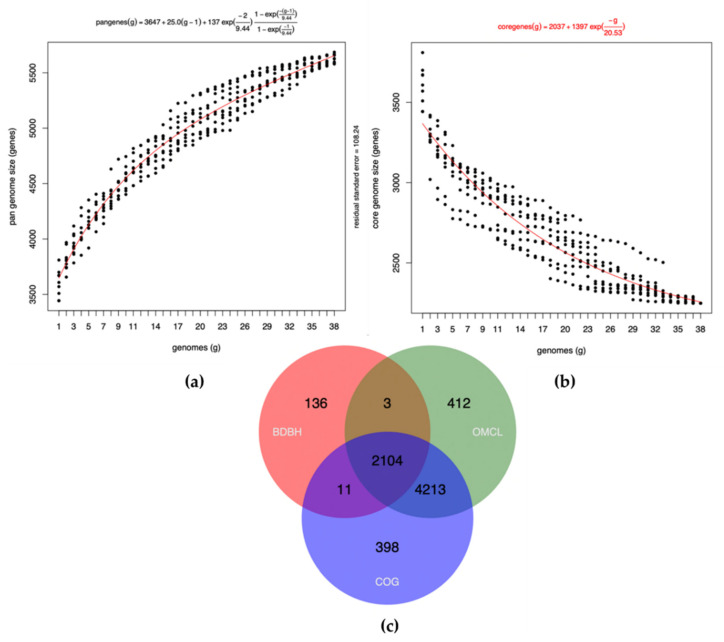
Plot of the estimation of pan- and core-genome sizes of *Bacillus pumilus* UAMX fitted with Tettelin function. (**a**) Pan-genome; (**b**) core-genome estimates; (**c**) Venn diagrams of core-genomes generated by BDBH, COG, and OMCL strategies.

Pan-genome includes the core and variable (or dispensable) genomes. The latter is comprised of genes shared by some strains of the same species as well as strain-specific genes. The BP-UAMX pan-genome (Figure 3a) showed a continuous increment (in genomes and gene families) as other *B. pumilus* genomes were incorporated, thereby indicating that the genome was still open.

**Figure 4:**
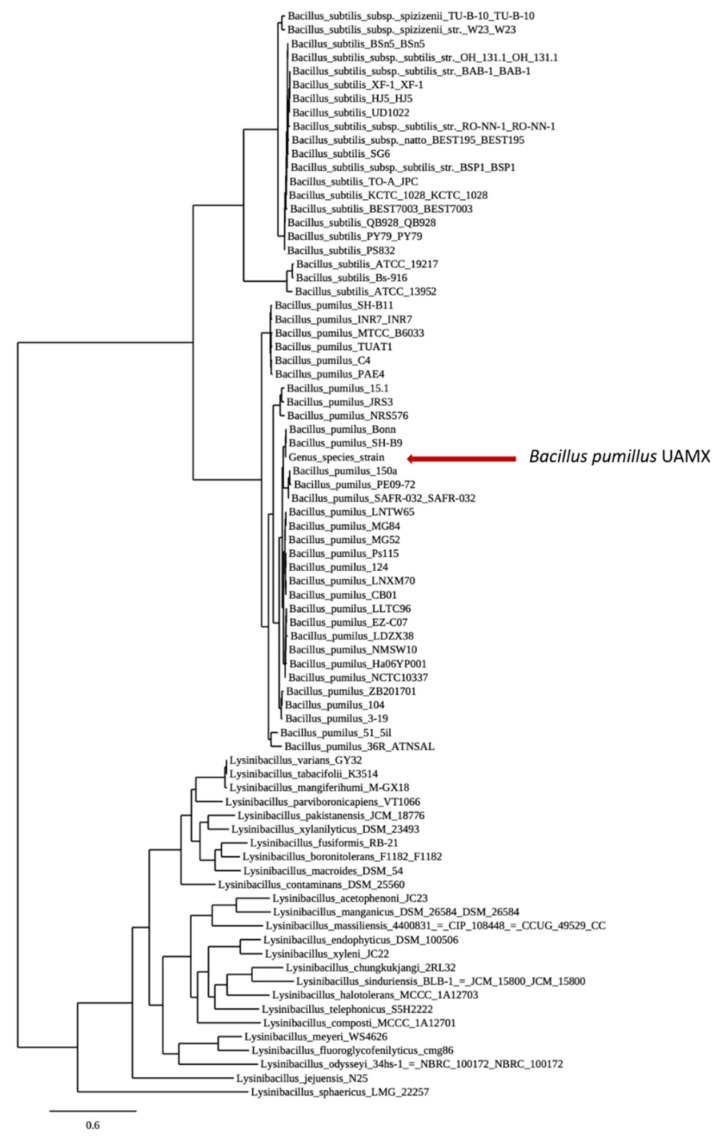
Pangenome tree for *B. pumilus* UAMX. Built with the closed genomes of *B. cereus*, *B. pumilus*, and genus *Lysinibacillus* strains.

An open pan-genome corresponds to a continuous increase in the number of genomes and gene families. In contrast, the core genomes diminished with the additional B. pumilus genomes (2104 essential genes, Figure 3b), which reflected the ability of the bacteria to obtain genetic material from other microorganisms of the same community, with the premise of global dispersibility [82]. Therefore, the open nature of BP-UAMX pan-genome is consistent with the hypothesis that bacterial species that inhabit a wide range of environments tend to possess an open pan-genome [61]. To the best of our knowledge, this was the first pan-genome analysis of a *B. pumilus* strain in which the core genomes were examined using BDBH, COGs, and OMCL strategies. The 2104 identified genes (Figure 3c) could thus represent the minimal set of critical genes that are essential for the survival of all *B. pumilus* analyzed in this study. Additionally, phylogenetic tree analysis (Figure 4) showed that BP-UAMX belonged to the *B. pumilus* group and was closely related to other strains isolated from human tissues, such as *B. pumilus* Bonn (Accession Number: LNCN00000000).

### 3.6. Gene Grouping by Function and Metabolic Pathway Prediction

Gene grouping according to the NCBI Clusters of Orthologous Groups (COGs) database (Table 2) indicated that a high percentage of genes were associated with general function prediction (8.81%) and metabolic function, such as amino acid metabolism and transport (9.8%), transcription (8.88%), translation, ribosomal structure, and biogenesis (7.25%), carbohydrate transport and metabolism (7.92%), and cell wall/membrane (5.76%). The predicted metabolic pathways were obtained using the Kyoto Encyclopedia of Genes and Genomes (KEGG) database. In general, most genes were associated with starch and sucrose metabolism, gluconeogenesis, tricarboxylic acid cycle, the pentose phosphate pathway, and pyruvate metabolism. Moreover, we identified genes that were associated with fatty acid degradation pathways, which agreed with the growth kinetics and protein profile results using tributyrin as a carbon source. Thus, BP-UAMX possesses genes encoding enzymes that allow the strain to metabolize alternative carbon sources to which it might be exposed in the GIT. Additionally, genes associated with the antibiotic resistance for vancomycin (*vanR-A*, *vanR-F*, *vanR-G*, *vanR-I*) and other antibiotics (*blaBPU*, *cat86*, *fosB*, *parY*, *sfrG*, and *suI4*) were identified using the Comprehensive Antibiotic Resistance database, even though only one beta-Lactam resistance protein related to the inhibition of peptidoglycan biosynthesis was detected in the proteomic approach (Table 1 and Supplementary Materials).

## 4. Conclusions

BP-UAMX isolated from the gastrointestinal tract of overweight individuals showed an adaptability to simulated GIT conditions, indicating that this strain of *B. pumilus* could survive in the gut. These findings reinforce the hypothesis that BP-UAMX could be a member of the GIT colonizing microbiota. Data from the growth kinetics, protein profile, gene grouping, and metabolic pathway prediction revealed that BP-UAMX could metabolize alternative carbon sources, such as starch, CMC, and lipids, that could be of great importance in the study of the GIT microbiota, which may be related to the effects of high-fat diets, since the production of monosaccharides and short chain fatty acids (SCFA) derived from the metabolism of GUT microbiota can provide additional energy to the host. Moreover, the host has benefits such as appetite and body mass control, which is provided by butyric acid.

This study represented a first approach to understanding the physiology of BP-UAMX. Although the purpose of this study was to assess the resistance of BP-UAMX to simulated gastric conditions, the genomic sequencing and bioinformatic analysis revealed alternative metabolic pathways in the UAMX strain that require further study.

## Figures and Tables

**Table 1:** Ontology of proteins expressed by *Bacillus pumilus* UAMX *.

Metabolic Pathway	Number of Proteins	
	Cytosol	Membrane
00010 Glycolysis/Gluconeogenesis	8	14
00020 Citrate cycle (TCA cycle)	3	11
00030 Pentose phosphate pathway	3	7
00051 Fructose and mannose metabolism	2	3
00052 Galactose metabolism	0	2
00061 Fatty acid biosynthesis	1	2
00071 Fatty acid degradation	1	1
00190 Oxidative phosphorylation	3	5
00220 Arginine biosynthesis	1	1
00261 Monobactam biosynthesis	1	1
00290 Valine, leucine, and isoleucine biosynthesis	1	2
00500 Starch and sucrose metabolism	0	3
00561 Glycerolipid metabolism	3	4
00564 Glycerophospholipid metabolism	1	2
00630 Glyoxylate and dicarboxylate metabolism	2	5
00790 Folate biosynthesis	1	1
00910 Nitrogen metabolism	1	1
00920 Sulfur metabolism	1	1
00983 Drug metabolism—other enzymes	2	2
01051 Biosynthesis of ansamycins	0	1
01501 beta-Lactam resistance	1	1
02010 ABC transporters	2	3
02024 Quorum sensing	3	6
02040 Flagellar assembly	2	2
02060 Phosphotransferase system (PTS)	2	3

* The completed ontology on the Supplementary Materials (ontology KASS KEGG analysis).

**Table 2:** Average COG categories and proteins of *Bacillus pumilus* UAMX.

Function	No. of Genes	%	No. of Cytosol Proteins	%	No. of Membrane Proteins	%
Chromatin structure and dynamics	1	0.03				
Energy production and conversion	159	5.06	11	9.09	29	10.36
Cell cycle control, cell division, chromosome partitioning	52	1.65	2	1.65	4	1.43
Amino acid transport and metabolism	308	9.80	6	4.96	19	6.79
Nucleotide transport and metabolism	85	2.70	9	7.44	15	5.36
Carbohydrate transport and metabolism	249	7.92	14	11.57	27	9.64
Coenzyme transport and metabolism	170	5.41	5	4.13	9	3.21
Lipid transport and metabolism	121	3.85	1	0.83	5	1.79
Translation, ribosomal structure, and biogenesis	228	7.25	30	24.79	56	20.00
Transcription	279	8.87	11	9.09	24	8.57
Replication, recombination, and repair	110	3.50	6	4.96	18	6.43
Cell wall/membrane/envelope biogenesis	181	5.76	1	0.83	4	1.43
Cell motility	61	1.94	0	0.00	0	0.00
Posttranslational modification, protein turnover, chaperones	118	3.75	11	9.09	16	5.71
Inorganic ion transport and metabolism	158	5.03	3	2.48	9	3.21
Secondary metabolites biosynthesis, transport, and catabolism	73	2.32	2	1.65	5	1.79
General function prediction only	277	8.81	1	0.83	10	3.57
Function unknown	183	5.82	2	1.65	5	1.79
Signal transduction mechanisms	177	5.63	4	3.31	15	5.36
Intracellular trafficking, secretion, and vesicular transport	27	0.86	1	0.83	2	0.71
Defense mechanisms	69	2.19	1	0.83	6	2.14
Extracellular structures	2	0.06	0	0.00	0	0.00
Mobilome: Prophages, transposons	56	1.78	0	0.00	2	0.71

## Data Availability

All data associated with this manuscript are given in the manuscript such as in the Supplementary Material the entire genome of BP-UAMX was sequenced and deposited in GenBank (NCBI) with the assigned accession number: “Bacillus pumilus str. UAMX isolate”: CP058951 (https://www.ncbi.nlm.nih.gov/nuccore/CP058951.1/, accessed on 12 April 2021).

## Supplementary Materials

The following are available online at https://www.mdpi.com/article/10.3390/microorganisms9051076/s1, Table S1: Ontology KASS KEGG analysis of *Bacillus pumilus* UAMX.
