# Supplementary material for: Physiological and Genomic Analysis of Bacillus pumilus UAMX Isolated from the Gastrointestinal Tract of Overweight Individuals
Source: Microorganisms. 2021 May 17;9(5):1076. doi: 10.3390/microorganisms9051076 (PMC8156450; doi:10.3390/microorganisms9051076)
Supplement: Supplementary file 1 [file microorganisms-09-01076-s001.zip › microorganisms-1205278-supplementary.pdf]

Table S1. Cytosol proteins. Microsoft Excel Ontology KASS KEGG analysis of *Bacillus pumilus* UAMX.

00010 Glycolysis / Gluconeogenesis (8)
00020 Citrate cycle (TCA cycle) (3)
00030 Pentose phosphate pathway (2)
00051 Fructose and mannose metabolism (2)
00053 Ascorbate and aldarate metabolism (1)
00061 Fatty acid biosynthesis (1)
00071 Fatty acid degradation (1)
00130 Ubiquinone and other terpenoid-quinone biosynthesis (1)
00190 Oxidative phosphorylation (3)
00195 Photosynthesis (2)
00220 Arginine biosynthesis (1)
00230 Purine metabolism (7)
00240 Pyrimidine metabolism (2)
00250 Alanine, aspartate and glutamate metabolism (3)
00260 Glycine, serine and threonine metabolism (1)
00261 Monobactam biosynthesis (1)
00270 Cysteine and methionine metabolism (3)
00280 Valine, leucine and isoleucine degradation (1)
00310 Lysine degradation (1)
00330 Arginine and proline metabolism (1)
00333 Prodigiosin biosynthesis (1)
00340 Histidine metabolism (1)
00380 Tryptophan metabolism (1)
00400 Phenylalanine, tyrosine and tryptophan biosynthesis (1)
00410 beta-Alanine metabolism (1)
00430 Taurine and hypotaurine metabolism (1)
00450 Selenocompound metabolism (5)
00480 Glutathione metabolism (1)
00520 Amino sugar and nucleotide sugar metabolism (2)
00561 Glycerolipid metabolism (3)
00564 Glycerophospholipid metabolism (1)
00620 Pyruvate metabolism (5)
00625 Chloroalkane and chloroalkene degradation (1)
00630 Glyoxylate and dicarboxylate metabolism (2)
00640 Propanoate metabolism (1)
00650 Butanoate metabolism (1)
00670 One carbon pool by folate (1)
00680 Methane metabolism (4)
00710 Carbon fixation in photosynthetic organisms (2)
00720 Carbon fixation pathways in prokaryotes (3)
00730 Thiamine metabolism (1)
00750 Vitamin B6 metabolism (1)
00760 Nicotinate and nicotinamide metabolism (1)
00770 Pantothenate and CoA biosynthesis (1)
00780 Biotin metabolism (1)
00790 Folate biosynthesis (1)
00860 Porphyrin and chlorophyll metabolism (1)
00903 Limonene and pinene degradation (1)
00910 Nitrogen metabolism (1)
00920 Sulfur metabolism (1)
00970 Aminoacyl-tRNA biosynthesis (9)
00981 Insect hormone biosynthesis (1)
00983 Drug metabolism - other enzymes (2)
01054 Nonribosomal peptide structures (1)
01501 beta-Lactam resistance (1)
02010 ABC transporters (2)
02020 Two-component system (2)
02024 Quorum sensing (3)
02040 Flagellar assembly (2)
02060 Phosphotransferase system (PTS) (2)
03010 Ribosome (10)
03018 RNA degradation (3)
03020 RNA polymerase (3)
03030 DNA replication (1)
03060 Protein export (1)
03070 Bacterial secretion system (1)

03320 PPAR signaling pathway (1)
03420 Nucleotide excision repair (1)
03440 Homologous recombination (1)
04066 HIF-1 signaling pathway (2)
04112 Cell cycle - Caulobacter (3)
04122 Sulfur relay system (1)
04212 Longevity regulating pathway - worm (2)
04217 Necroptosis (1)
04626 Plant-pathogen interaction (2)
04724 Glutamatergic synapse (1)
04727 GABAergic synapse (1)
04922 Glucagon signaling pathway (1)
04930 Type II diabetes mellitus (1)
04931 Insulin resistance (1)
04940 Type I diabetes mellitus (1)
05010 Alzheimer disease (1)
05130 Pathogenic Escherichia coli infection (1)
05132 Salmonella infection (1)
05134 Legionellosis (2)
05152 Tuberculosis (2)
05165 Human papillomavirus infection (1)
05203 Viral carcinogenesis (1)
05230 Central carbon metabolism in cancer (1)
05415 Diabetic cardiomyopathy (2)
05417 Lipid and atherosclerosis (1)

Table S2. Membrane proteins. Cytosol proteins. Microsoft Excel Ontology KASS KEGG analysis of *Bacillus pumilus* UAMX.

00010 Glycolysis / Gluconeogenesis (14)
00020 Citrate cycle (TCA cycle) (11)
00030 Pentose phosphate pathway (7)
00040 Pentose and glucuronate interconversions (1)
00051 Fructose and mannose metabolism (3)
00052 Galactose metabolism (2)
00053 Ascorbate and aldarate metabolism (1)
00061 Fatty acid biosynthesis (2)
00071 Fatty acid degradation (1)
00130 Ubiquinone and other terpenoid-quinone biosynthesis (1)
00190 Oxidative phosphorylation (5)
00195 Photosynthesis (2)
00220 Arginine biosynthesis (1)
00230 Purine metabolism (9)
00240 Pyrimidine metabolism (5)
00250 Alanine, aspartate and glutamate metabolism (4)
00260 Glycine, serine and threonine metabolism (6)
00261 Monobactam biosynthesis (1)
00270 Cysteine and methionine metabolism (4)
00280 Valine, leucine and isoleucine degradation (3)
00290 Valine, leucine and isoleucine biosynthesis (2)
00310 Lysine degradation (3)
00330 Arginine and proline metabolism (1)
00333 Prodigiosin biosynthesis (1)
00340 Histidine metabolism (1)
00380 Tryptophan metabolism (3)
00400 Phenylalanine, tyrosine and tryptophan biosynthesis (2)
00410 beta-Alanine metabolism (1)
00430 Taurine and hypotaurine metabolism (2)
00450 Selenocompound metabolism (5)
00480 Glutathione metabolism (3)
00500 Starch and sucrose metabolism (3)
00520 Amino sugar and nucleotide sugar metabolism (4)
00541 O-Antigen nucleotide sugar biosynthesis (1)
00561 Glycerolipid metabolism (4)
00564 Glycerophospholipid metabolism (2)
00620 Pyruvate metabolism (12)
00625 Chloroalkane and chloroalkene degradation (1)
00630 Glyoxylate and dicarboxylate metabolism (5)
00640 Propanoate metabolism (9)
00650 Butanoate metabolism (3)
00660 C5-Branched dibasic acid metabolism (3)
00670 One carbon pool by folate (1)
00680 Methane metabolism (6)
00710 Carbon fixation in photosynthetic organisms (3)
00720 Carbon fixation pathways in prokaryotes (9)
00730 Thiamine metabolism (1)
00750 Vitamin B6 metabolism (1)
00760 Nicotinate and nicotinamide metabolism (2)
00770 Pantothenate and CoA biosynthesis (3)
00780 Biotin metabolism (1)
00785 Lipoic acid metabolism (1)
00790 Folate biosynthesis (1)
00860 Porphyrin and chlorophyll metabolism (2)

00903 Limonene and pinene degradation (1)
00910 Nitrogen metabolism (1)
00920 Sulfur metabolism (1)
00970 Aminoacyl-tRNA biosynthesis (11)
00981 Insect hormone biosynthesis (1)
00983 Drug metabolism - other enzymes (2)
01051 Biosynthesis of ansamycins (1)
01054 Nonribosomal peptide structures (1)
01501 beta-Lactam resistance (1)
02010 ABC transporters (3)
02020 Two-component system (8)
02024 Quorum sensing (6)
02040 Flagellar assembly (2)
02060 Phosphotransferase system (PTS) (3)
03010 Ribosome (19)
03013 RNA transport (1)
03018 RNA degradation (6)
03020 RNA polymerase (3)
03030 DNA replication (2)
03060 Protein export (2)
03070 Bacterial secretion system (2)
03320 PPAR signaling pathway (1)
03420 Nucleotide excision repair (2)
03430 Mismatch repair (3)
03440 Homologous recombination (3)
04066 HIF-1 signaling pathway (6)
04112 Cell cycle - Caulobacter (4)
04122 Sulfur relay system (1)
04146 Peroxisome (1)
04152 AMPK signaling pathway (1)
04212 Longevity regulating pathway - worm (2)
04217 Necroptosis (1)
04626 Plant-pathogen interaction (2)
04724 Glutamatergic synapse (1)
04727 GABAergic synapse (1)
04919 Thyroid hormone signaling pathway (1)
04922 Glucagon signaling pathway (5)
04930 Type II diabetes mellitus (1)
04931 Insulin resistance (1)
04940 Type I diabetes mellitus (1)
05010 Alzheimer disease (1)
05130 Pathogenic Escherichia coli infection (1)
05132 Salmonella infection (1)
05134 Legionellosis (3)
05152 Tuberculosis (2)
05165 Human papillomavirus infection (1)
05203 Viral carcinogenesis (1)
05230 Central carbon metabolism in cancer (7)
05415 Diabetic cardiomyopathy (5)
05417 Lipid and atherosclerosis (1)
